# Thyroid Hormone Receptor β1 and PGC1α Coordinately Regulate OPA1/MFN2‐Mediated Mitochondrial Fusion and UCP1‐Mediated Lipid Browning in ccRCC

**DOI:** 10.1002/advs.202508571

**Published:** 2025-11-09

**Authors:** Xiangui Meng, Tiexi Yu, Fang Lv, Weiquan Li, Hongmei Yang, Xiaoping Zhang, Wen Xiao

**Affiliations:** ^1^ Department of Urology Union Hospital Tongji Medical College Huazhong University of Science and Technology Wuhan 430022 China; ^2^ Shenzhen Huazhong University of Science and Technology Research Institute Shenzhen 518000 China; ^3^ Institute of Urology Tongji Medical College Huazhong University of Science and Technology Wuhan 430022 China; ^4^ Department of Pathogenic Biology School of Basic Medicine Tongji Medical College Huazhong University of Science and Technology Wuhan 430030 China

**Keywords:** ccRCC, lipid browning, mitochondrial fusion, the peroxisome proliferator‐activated receptor gamma coactivator 1‐alpha (PGC1α), the thyroid hormone receptor β1 (TRβ)

## Abstract

The abnormal accumulation of lipids is a hallmark of clear cell renal cell carcinoma (ccRCC). Both the thyroid hormone receptor β1 (TRβ) and peroxisome proliferator‐activated receptor gamma coactivator 1‐alpha (PGC1α) are key regulators of mitochondrial function and lipid metabolism. However, their specific interaction and influence on ccRCC development and lipid accumulation remain poorly understood. This study identified genes jointly regulated by TRβ and PGC1α, which are implicated in lipid browning and mitochondrial fusion. Mechanistically, T3‐activated TRβ interacts with PGC1α to transcriptionally upregulate PGC1α, UCP1, and mitochondrial fusion genes OPA1 and MFN2, thereby enhancing mitochondrial activity, promoting lipid utilization, and suppressing ccRCC progression. These results indicate that the mitochondrial and metabolic effects of TRβ in ccRCC are mediated through PGC1α expression and function. Activation of the TRβ/PGC1α through hormonal and pharmacological means may offer a promising therapeutic approach for ccRCC.

## Graphical abstract

Thyroid hormone receptor β1 (TRβ) downregulation correlates with poor prognosis in clear cell renal cell carcinoma (ccRCC). Restoration of TRβ and the peroxisome proliferator‐activated receptor gamma coactivator 1‐alpha suppresses tumor progression in ccRCC through Uncoupling protein 1 mediated tumor cell “slimming” and optic atrophy 1/mitofusin 2 dependent mitochondrial fusion.

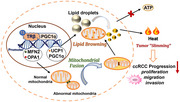

## 1 Introduction

The thyroid hormone receptor β (THRB), a triiodothyronine (T3)‐dependent transcription factor, is essential for development, growth, differentiation, and metabolic homeostasis. Although substantial progress has been made in understanding the molecular mechanisms of THRB in maintaining normal cellular functions, its role in carcinogenesis remains largely elusive. Mutations in THRB have been identified in several cancers, including hepatocellular carcinoma, breast cancer, pituitary tumors, and thyroid cancer, implicating its involvement in tumorigenesis. In addition, loss of THRB expression due to truncation or deletion on chromosome 3p has been observed in cervical, ovarian, and testicular cancers, suggesting a potential tumor‐suppressive function.^[^ ^1^ ^]^ The THRB gene encodes two primary receptor subtypes, THRB1 and THRB2. THRB1 (TRβ) is predominantly expressed in the liver and kidney, whereas THRB2 expression is largely restricted to the hypothalamus and pituitary.^[^ ^2^ ^]^ Although TRβ downregulation has been documented in renal carcinoma,^[^ ^3^ ^]^ its precise biological function in this context remains unclear.

Clear cell renal cell carcinoma (ccRCC) is widely recognized as a metabolic disorder,^[^ ^4^ ^]^ primarily characterized by abnormal lipid accumulation.^[^ ^5^ ^]^ Lipid browning, which converts white adipose tissue into brown adipose tissue (BAT) or “beige” fat, is a key mechanism that links energy expenditure with lipid metabolism. By promoting the browning of white fat and enhancing lipid oxidation, this process offers potential control over obesity and related metabolic disorders. Uncoupling protein 1 (UCP‐1), exclusively expressed in brown adipocytes, promotes lipid consumption without generating ATP, thereby reducing lipid accumulation and contributing to body weight regulation.^[^ ^6^ ^]^ Consequently, understanding the mechanisms that drive UCP‐1 upregulation is crucial for addressing obesity and related comorbidities.^[^ ^7^, ^8^ ^]^ Although the regulation of UCP‐1 has been extensively studied in the context of obesity and diabetes, its role in cancer biology remains largely unexplored. Our previous study highlighted lipid browning as a novel mechanism in ccRCC tumorigenesis, proposing a therapeutic approach termed “tumor slimming.” By modulating key lipid browning genes, such as PGC1α and UCP1, lipid autophagy is induced within tumor cells, initiating browning and reducing tumor size and volume.^[^ ^9^, ^10^ ^]^ Furthermore, nicotinamide nucleotide transhydrogenase enhances lipid browning and counteracts the tumor‐promoting effects of HIF2α by reducing lipid deposition.^[^ ^11^ ^]^ Recent studies have focused on the role of the T3/TRβ axis in enhancing heat production and maintaining lipid homeostasis,^[^ ^12^ ^]^ particularly through the promotion of lipid browning.^[^ ^13^ ^]^ Evidence also shows that T3‐activated TRβ induces UCP1‐mediated browning in vitro, while TR agonist GC‐1 promotes browning in white adipocytes and triggers a BAT‐like thermogenic response in genetically obese mice.^[^ ^14^ ^]^ This process—characterized by increased metabolism, fat loss, and improved cold tolerance—suggests that lipid browning may play a previously underappreciated role in TRβ‐induced thermogenesis and its potential implications in oncology. Nevertheless, whether TRβ modulates aberrant lipid metabolism in ccRCC via lipid browning pathways remains unclear.

Mitochondria, the cellular powerhouse, function as the central hub for thermogenesis and energy metabolism, integrating key processes such as energy generation, metabolic regulation, apoptosis, and innate immune responses.^[^ ^15^, ^16^, ^17^, ^18^ ^]^ Mitochondrial defects lead to metabolic alterations, including increased glycolysis and abnormal lipid reprogramming.^[^ ^19^, ^20^ ^]^ Recent studies indicate that mitochondrial morphology in cells is highly dynamic, undergoing continuous remodeling through fusion, fission, and mitophagy.^[^ ^21^, ^22^, ^23^ ^]^ These dynamic processes are critical for maintaining mitochondrial integrity, function, and particularly energy supply.^[^ ^24^ ^]^ Moreover, impaired mitochondrial dynamics contribute significantly to neurodegenerative diseases and cancer progression by disrupting apoptosis, energy production, and signal transduction. Investigating the regulatory mechanisms that govern these processes will greatly aid in identifying therapeutic targets for cancer. Mitochondrial fusion is a two‐step process involving outer membrane fusion mediated by mitofusin 1/2 (MFN1/2), followed by inner membrane fusion facilitated by optic atrophy 1 (OPA1).^[^ ^25^, ^26^ ^]^ The MFN2 R707W mutation, in particular, is associated with multiple symmetric lipomatosis, a condition characterized by abnormal fat accumulation and metabolic disturbances, including low leptin levels, hypertriglyceridemia, insulin resistance, diabetes, and lipid metabolism.^[^ ^27^, ^28^, ^29^, ^30^ ^]^ In hepatocellular carcinoma, suppression of MFN1‐dependent mitochondrial fusion enhances tumorigenesis.^[^ ^31^ ^]^ Notably, mitochondrial dynamics are closely linked to bioenergetics, as exemplified by the promotion of mitochondrial fusion and elongation under conditions of elevated oxidative phosphorylation. However, whether TRβ can directly modulate OPA1/MFN2‐mediated mitochondrial fusion, thereby impacting lipid metabolic remodeling, remains unclear.

This study demonstrates that T3‐activated TRβ enhances the transcription of UCP1 and mitochondrial fusion genes OPA1 and MFN2 through its interaction with the coactivator PGC1α. This interaction promotes mitochondrial fusion and function, thereby enhancing lipid consumption and suppressing tumor progression in ccRCC. The availability of selective pharmacological agonists targeting TRβ and PGC1α presents an opportunity to further investigate the underlying mechanisms of these pathways and explore potential therapeutic strategies for metabolic diseases and cancer.

## 2 Results

### 2.1 Restoration of TRβ Activation in ccRCC Cells Inhibits Lipid Accumulation and Tumor Progression

**Figure 1:**
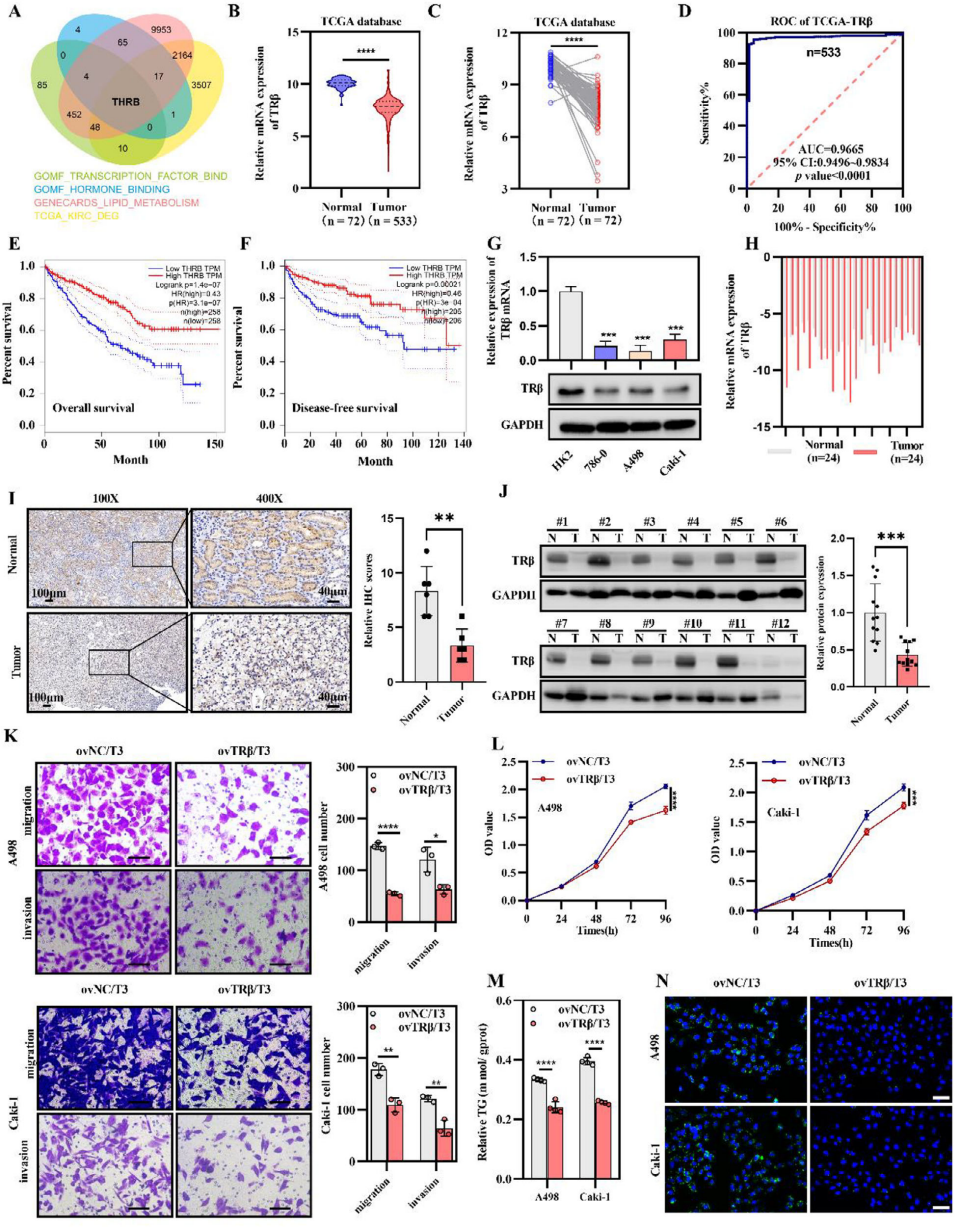
Activation of TRβ in ccRCC cells reduces lipid accumulation and inhibits cancer progression. A) Venn diagram showing differentially expressed genes from the Cancer Genome Atlas‐Kidney Renal Clear Cell Carcinoma (TCGA‐KIRC) and three MsigDB datasets (https://www.gsea‐msigdb.org/gsea/msigdb). B) TRβ mRNA expression in ccRCC tissues from the TCGA‐KIRC database. C) Comparison of TRβ mRNA levels between ccRCC and matched adjacent normal tissues from the TCGA‐KIRC dataset. D) Receiver Operating Characteristic (ROC) curve analysis of TRβ expression using TCGA‐KIRC data. E) Kaplan‒Meier survival curve assessing overall survival in patients with ccRCC based on TRβ expression (log‐rank test). F) Kaplan‒Meier analysis of disease‐free survival in relation to TRβ expression in patients with ccRCC (log‐rank test). G) TRβ mRNA and protein expression in ccRCC cell lines compared to normal cells (*n* = 3). H) TRβ mRNA levels in ccRCC tissues versus adjacent normal tissues from 24 patients (*t*‐test). I) Representative immunohistochemical images showing TRβ expression in ccRCC and adjacent normal tissues; dark brown staining indicates strong positivity. Statistical analysis was performed on six randomly selected fields. J) Comparison of TRβ protein levels and statistical charts between ccRCC and paired adjacent normal tissues (*n* = 12). K) Transwell assay results comparing the migration of TRβ‐overexpressing cells with control cells in the presence of T3 (*n* = 3). L) CCK8 assay showing proliferation of TRβ‐overexpressing cells treated with T3 (*n* = 4). M) Triglyceride (TG) concentration (mmol g^−1^ protein) in TRβ‐overexpressing cells measured by TG assay (*n* = 3, *t*‐test). N) Bodipy staining showing lipid content in TRβ‐overexpressing and control cell lines (*n* = 3, *t*‐test). Data represent a minimum of three independent experiments (*p < 0.05, **p < 0.01, ***p < 0.001). TRβ overexpression lentivirus (ovTRβ); negative controls (ovNC).

To investigate the mechanisms underlying metabolic abnormalities in ccRCC, we analyzed four datasets consisting of genes associated with lipid metabolism, hormone signaling, transcriptional regulation, and differentially expressed genes from the TCGA‐KIRC database. Unexpectedly, TRβ emerged as a potential central regulator of ccRCC progression (**Figure** **1**A). Specifically, TRβ mRNA expression was significantly lower in ccRCC tissues than in both the total cohort of 533 tumor samples and 72 paired tumor‐normal tissues (Figure 1B,C). Receiver operating characteristic (ROC) curve analysis demonstrated a high clinical diagnostic value for TRβ, with an AUC of 0.9665 (total cohort; 95% CI: 0.9496‒0.9839; *p* < 0.0001) (Figure 1D) and 0.9792 (paired samples; 95% CI: 0.9525‒1.000; *p* < 0.0001) (Figure S1A, Supporting Information). Kaplan‒Meier survival curves further indicated a positive correlation between TRβ expression and both overall (Figure 1E) and disease‐free (Figure 1F) survivals in patients with ccRCC.

The downregulation of TRβ at the mRNA and protein levels in ccRCC was confirmed in cell lines (Figure 1G) and further validated in tumor tissues by immunohistochemistry (IHC) and western blotting, consistent with bioinformatics findings (Figure 1H–J; Figure S1B, Supporting Information). In addition, TRβ downregulation was correlated with advanced tumor, node, and metastasis staging and higher G grades in ccRCC (**Table** **1**). Univariate and multivariate Cox regression analyses revealed that reduced TRβ expression is an independent unfavorable prognostic factor for patients with ccRCC, even after adjusting for established clinical risk factors (**Table** **2**).

**Table 1:** Correlation between THRB mRNA expression and clinicopathological parameters of ccRCC patients.

Parameter		Total	THRB mRNA expression		*p*
			Low (*n* = 265)	High (*n* = 265)	
Age (years)	≤60	263	132	131	0.931
	>60	267	133	134	
Gender	male	342	167	1175	0.468
	female	188	98	90	
T stage	T1 + T2	339	149	190	0.000*
	T3 + T4	191	116	75	
N stage	Nx + N0	513	252	261	0.046*
	N1	17	13	4	
M stage	Mx + M0	452	216	236	0.014*
	M1	78	49	19	
TNM stage	I + II	321	135	186	0.000*
	III + IV	209	130	79	
G grade	Gx + G1 + G2	247	99	148	0.000*
	G3 + G4	283	166	117	

“*” indicates that the *p*‐value is statistically significant, *p* < 0.05.

Relevant clinical data of ccRCC patients are all from the TCGA‐KIRC database.

**Table 2:** Univariate and multivariate Cox analyses of overall survival of ccRCC patients.

Parameter		Total	Univariate analysis			Multivariate analysis		
			HR	95% CI	*p*	HR	95% CI	*p*
Age (years)	<60	247	1.747	1.288–2.369	0.000*	1.677	1.234–2.278	0.001*
	≥60	283						
Gender	Male	347	0.944	0.695–1.283	0.713			
	Female	183						
T stage	T1 + T2	340	3.159	2.337–4.271	0.000*	0.910	0.502–1.650	0.757
	T3 + T4	190						
N stage	Nx + N0	514	4.037	2.239–7.282	0.000*	2.061	1.121–3.788	0.020*
	N1	16						
M stage	Mx + M0	450	4.251	3.120–5.791	0.000*	2.322	1.602–3.365	0.000*
	M1	78						
TNM stage	I + II	322	3.858	2.816–5.285	0.000*	2.149	1.084–4.257	0.028*
	III + IV	205						
G Grade	Gx + G1 + G2	248	2.636	1.883–3.691	0.000*	1.587	1.106–2.277	0.012*
	G3 + G4	279						
THRB	Low	265 265	0.497	0.365–0.676	0.000*	0.659	0.480–0.906	0.010*
	High							

“*” indicates that the *p*‐value is statistically significant, p < 0.05.

Relevant clinical data of ccRCC patients are all from the TCGA‐KIRC database.

Given the substantial reduction of TRβ expression in ccRCC, TRβ‐specific Caki‐1 and A498 overexpressing cell lines were generated (Figure S1C–E, Supporting Information) to assess the biological impact of TRβ restoration on ccRCC malignancy. Notably, neither TRβ overexpression alone nor T3 stimulation significantly altered ccRCC malignant progression (Figure S1F,G, Supporting Information). However, upon T3 activation, TRβ overexpression significantly suppressed proliferation, migration, and invasion in ccRCC cells (Figure 1K,L). Consistent with previous studies that highlighted impaired lipid metabolism and aberrant lipid droplet accumulation in ccRCC, this study observed a marked reduction in triglyceride content in ccRCC cells following enhanced TRβ activation (Figure 1M). Notably, TRβ overexpression induced a pronounced “slimming” effect in tumor cells. Relative fluorescence intensity (bodipy) staining revealed a decreased lipid accumulation in TRβ‐restored Caki‐1 and A498 cell lines (Figure 1N; Figure S1H, Supporting Information). In summary, our findings provide compelling evidence that TRβ acts as a tumor suppressor in ccRCC. Restoration of TRβ activation effectively inhibits lipid accumulation and tumor progression in vitro, underscoring its potential as a promising therapeutic target in ccRCC.

### 2.2 TRβ Reactivation Promotes PGC1α/UCP1‐Mediated Lipid Browning and Tumor Cell “Slimming” in ccRCC

**Figure 2:**
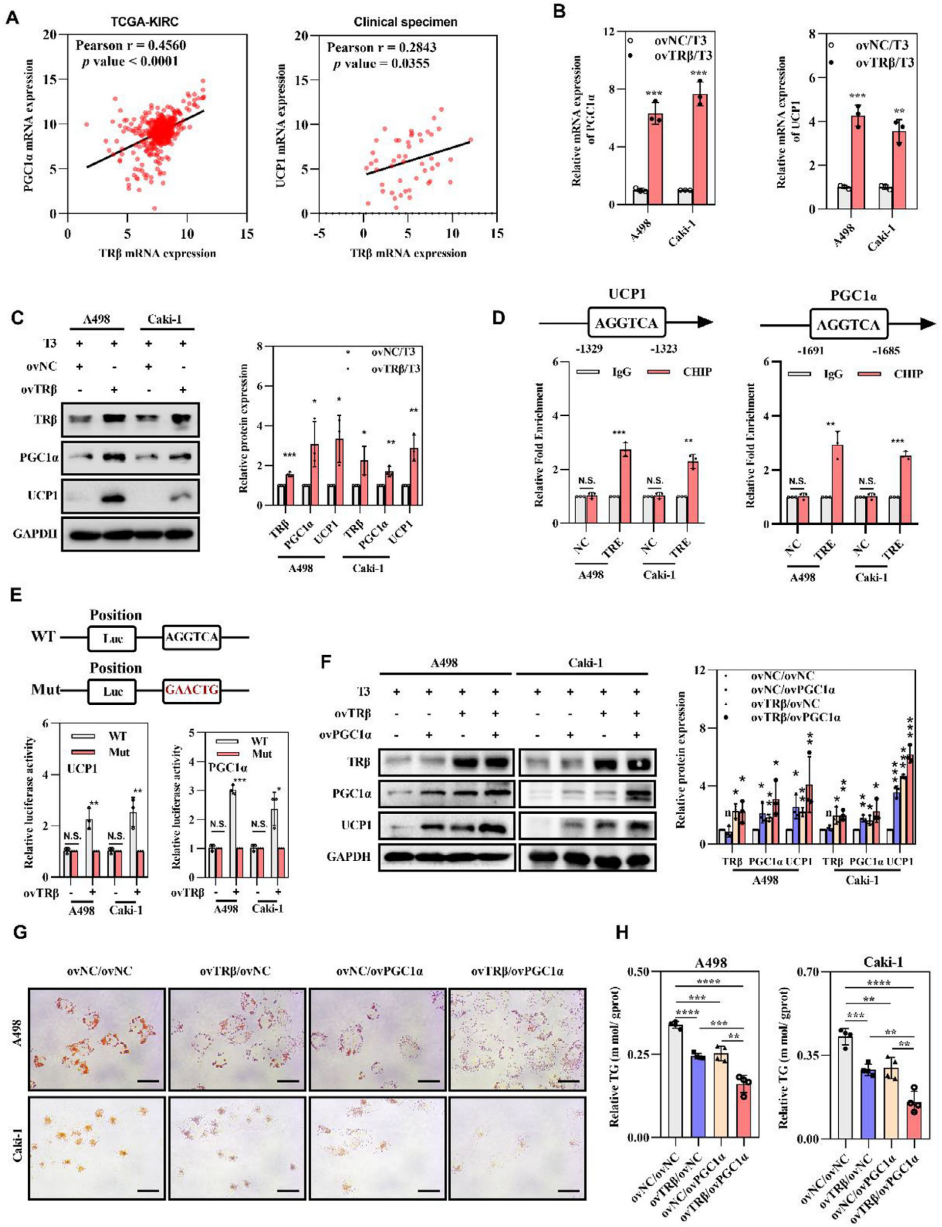
TRβ reactivation promotes lipid browning through the PGC1α/UCP1 pathway, leading to tumor cell “slimming” in ccRCC. A) Correlation analysis between TRβ and PGC1α in the TCGA‐KIRC dataset and ccRCC clinical tissues. B) PGC1α mRNA levels in TRβ‐overexpressing ccRCC cell lines versus controls following Triiodothyronine (T3) treatment (*n* = 3). C) Protein levels of PGC1α and UCP1 in TRβ‐overexpressing cell lines compared to controls after T3 treatment (*n* = 3). D) Chromatin immunoprecipitation followed by quantitative PCR (ChIP‐PCR) results in ccRCC cell lines (*n* = 3). E) Dual‐luciferase reporter assay data in ccRCC cells (*n* = 3). F) Protein expression of PGC1α and UCP1 in treated cell lines (*n* = 3). G) Oil Red O staining of selected cell lines. H) Triglyceride (TG) levels in the designated cell lines (*n* = 3). Data represent at least three independent experiments (*p < 0.05, **p < 0.01, ***p < 0.001). TRβ, PGC1α overexpression lentivirus (ovTRβ, ovPGC1α); negative controls (ovNC).

Previous studies have demonstrated that PGC1α inhibits tumor progression and reduces lipid accumulation through UCP1‐mediated lipid browning‐like mechanisms.^[^ ^10^ ^]^ To further investigate whether TRβ contributes to lipid reduction via similar pathways, a correlation heatmap and linear regression analysis were performed to evaluate the potential relationships between TRβ, PGC1α, and UCP1. A positive correlation was identified between TRβ and PGC1α (Pearson r = 0.46, *p* < 0.001), as well as between TRβ and UCP1 mRNA levels (Pearson r = 0.2843, *p* = 0.0355), based on analyses of the TCGA dataset and clinical samples (**Figure** **2**A). Further qPCR and western blot analyses revealed that T3/TRβ reactivation significantly elevated both mRNA and protein levels of PGC1α and UCP1 (Figure 2B,C). To elucidate the mechanism by which TRβ regulates PGC1α and UCP1 transcription, a thyroid hormone receptor response element (TRE; AGGTCA) was identified within the proximal promoters of both target genes. Chromatin immunoprecipitation (ChIP) followed by qPCR confirmed that TRβ directly binds to the TRE regions within the PGC1α and UCP1 promoters (Figure 2D). Luciferase reporter assays further demonstrated a substantial increase in PGC1α and UCP1 promoter activities following transfection with wild‐type TRβ, whereas mutated TRβ failed to produce a similar effect (Figure 2E). These results confirm that TRβ directly activates PGC1α and UCP1 expression by binding to TREs in their proximal promoters.

Subsequently, we examined the role of TRβ reactivation in modulating PGC1α/UCP1‐mediated lipid browning and tumor cell “slimming” in ccRCC. Restoration of PGC1α alone upregulated UCP1 expression, and overexpression of TRβ further amplified UCP1 gene transactivation and protein levels (Figure 2F). Reactivation of either PGC1α or TRβ independently reduced lipid accumulation in ccRCC cells, whereas co‐restoration of both produced the most significant reduction in lipid deposition, as confirmed by Oil Red O staining and triglyceride content measurements (Figure 2G,H). Collectively, these results highlight that TRβ reactivation enhances PGC1α/UCP1‐mediated lipid browning, reinforcing its potential role in inhibiting lipid accumulation and tumor progression in ccRCC.

### 2.3 Restoration of TRβ and PGC1α Inhibits Tumor Progression in ccRCC In Vivo and In Vitro

**Figure 3:**
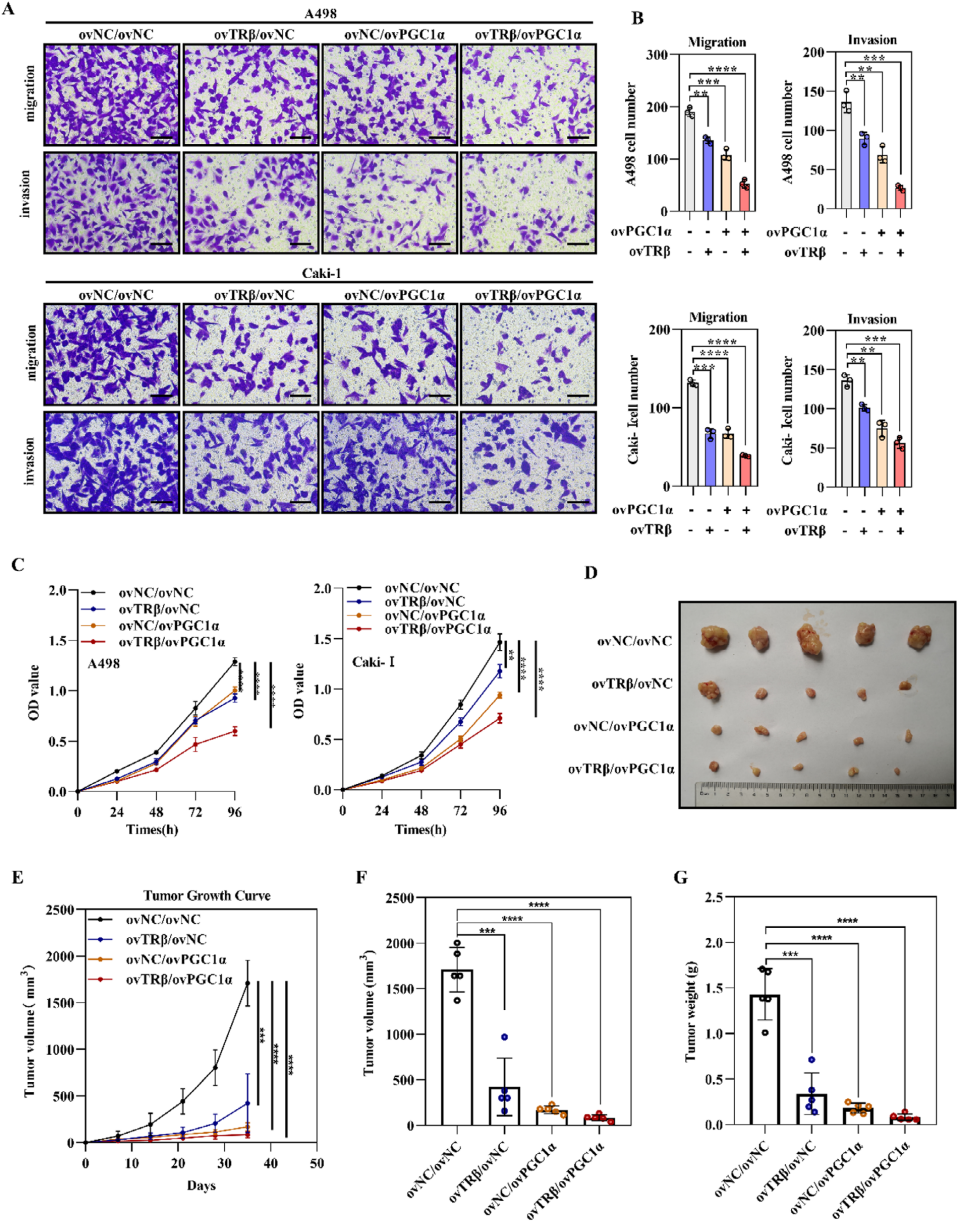
Restoration of TRβ and PGC1α inhibits tumor growth in ccRCC in both in vitro and in vivo models. A,B) Transwell migration assays assessing the metastatic potential of ccRCC cells expressing TRβ and/or PGC1α (*n* = 3). C) Cell proliferation measured by CCK‐8 assays in RCC cells overexpressing TRβ and/or PGC1α (*n* = 4). D) Representative images of tumors derived from A498 cells with TRβ and/or PGC1α overexpression in xenograft models (*n* = 5). E) Tumor growth curves for xenografts across the four experimental groups (*n* = 5). F) Tumor volumes in xenograft models across the four groups (*n* = 5). G) Tumor weights in xenograft models for each group (*n* = 5). Data represent at least three independent experiments (*p < 0.05, **p < 0.01, ***p < 0.001). TRβ, PGC1α overexpression lentivirus (ovTRβ, ovPGC1α); negative controls (ovNC).

The effects of TRβ and PGC1α restoration on lipid browning and tumor cell “slimming” were further assessed through in vivo and in vitro studies. Transwell assays demonstrated that PGC1α restoration significantly reduced the migratory and invasive capacities of ccRCC cell lines, and TRβ overexpression further amplified this inhibitory effect (**Figure** **3**A,B). In addition, CCK8 cell viability assays indicated that TRβ overexpression notably intensified the PGC1α‐induced suppression of ccRCC proliferation (Figure 3C). These findings indicate that TRβ and PGC1α act synergistically to impede tumor progression in vitro.

To validate these in vitro findings, Caki‐1 cells overexpressing TRβ and/or PGC1α were subcutaneously injected into BALB/c nude mice. The restoration of either TRβ or PGC1α individually exerted anti‐tumor effects, reducing both the volume and weight of the xenografts. Furthermore, the combined overexpression of TRβ and PGC1α led to a synergistic enhancement of tumor inhibition (Figure 3D–G). Collectively, these results demonstrate that TRβ restoration potentiates the PGC1α‐mediated suppression of tumor progression in ccRCC in vitro and in vivo.

### 2.4 TRβ Repressed ccRCC Progression and Promoted Tumor Cell “Slimming” in a PGC1α‐Depended Manner

**Figure 4:**
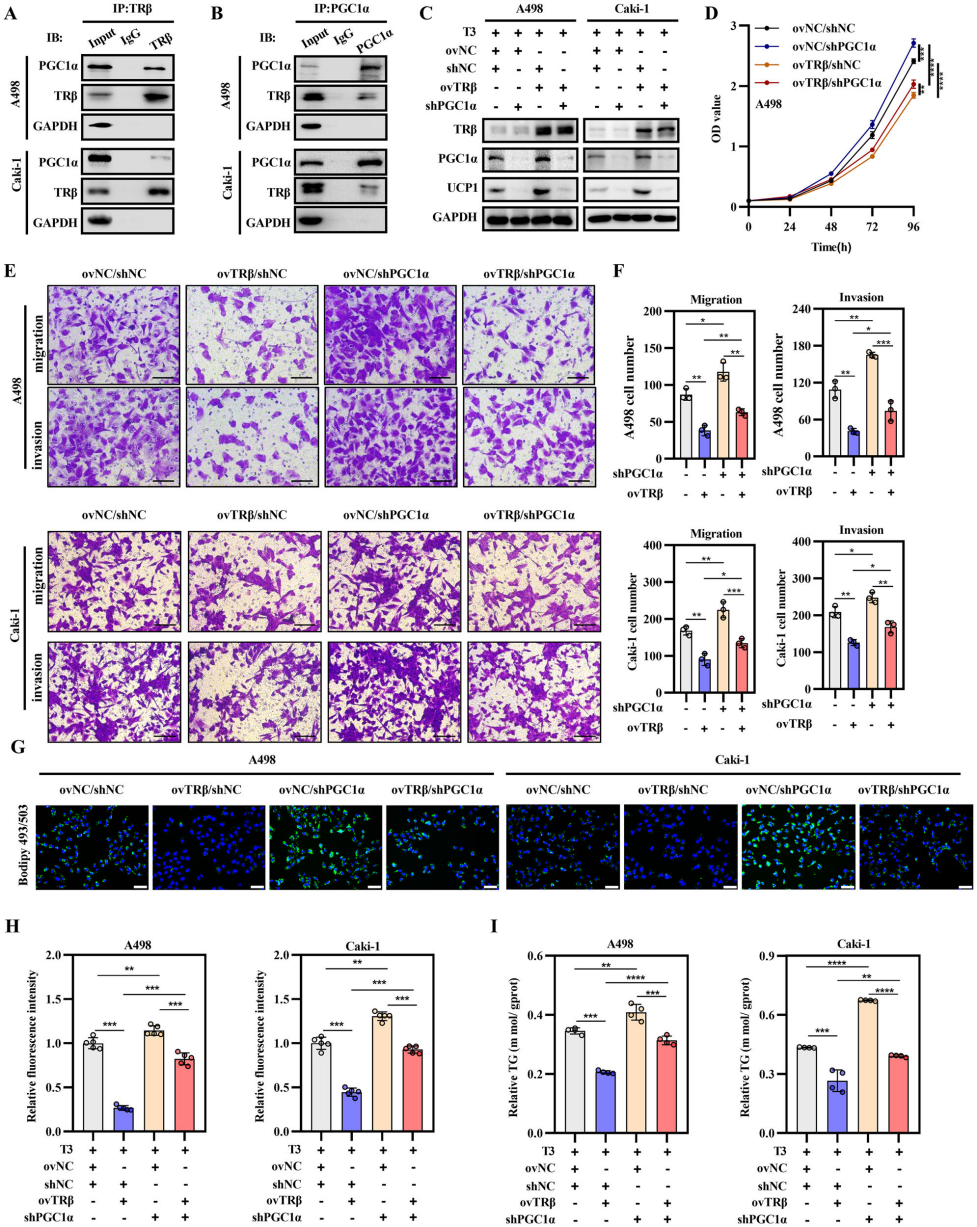
TRβ suppresses ccRCC progression and induces tumor “slimming” in a PGC1α‐dependent manner. A,B) Co‐immunoprecipitation (Co‐IP) assays confirmed the interaction between TRβ and PGC1α in ccRCC cell lines. C) Western blotting analysis of TRβ and PGC1α protein levels in ccRCC cells with TRβ overexpression and/or PGC1α knockdown. D) Cell growth assessed using CCK8 assays in TRβ‐overexpressing cells with or without PGC1α knockdown (*n* = 4). E,F) Transwell assays evaluating the metastatic potential of ccRCC cells expressing TRβ and/or PGC1α knockdown (*n* = 3). G,H) Bodipy staining was performed to assess lipid droplet accumulation in ccRCC cells overexpressing TRβ and/or with PGC1α knockdown; statistical analysis was performed using a *t*‐test. I) Triglyceride assay in the same experimental groups. Data represent at least three independent experiments (*p < 0.05, **p < 0.01, ***p < 0.001). TRβ overexpression lentivirus (ovTRβ); PGC1α knockout lentivirus (sh‐PGC1α); negative controls (ovNC).

PGC‐1α has been established as a potent transcriptional coactivator for TRβ.^[^ ^32^ ^]^ However, the interaction between TRβ and PGC1α in UCP1‐mediated lipid browning within ccRCC remains unclear. Immunoprecipitation with anti‐TRβ followed by western blotting with anti‐PGC1α confirmed the interaction between TRβ and PGC1α in co‐transfected cell extracts (**Figure** **4**A). Reciprocal immunoprecipitation using anti‐PGC1α and western blotting with anti‐TRβ produced similar results, indicating that PGC1α directly interacts with reactivated TRβ (Figure 4B). These results suggest that the tumor‐suppressive effects of TRβ/PGC1α in ccRCC may depend on their collaborative activation. To test this hypothesis, A498 and Caki‐1 cells with stable TRβ overexpression were engineered with PGC1α knockdown using shRNA. As expected, PGC1α knockdown resulted in decreased UCP1 levels and partially reversed the TRβ‐induced upregulation of UCP1 (Figure 4C).

Functional rescue experiments were subsequently conducted to determine whether TRβ exerts its tumor‐suppressive effects in ccRCC through a PGC1α‐dependent mechanism. The results showed that PGC1α knockdown increased the growth, migration, and invasion of A498 and Caki‐1 cells, while negating the inhibitory effects of TRβ restoration on RCC malignancy (Figure 4D–F). In addition, bodipy and Oil Red O staining revealed a marked increase in lipid accumulation following PGC1α knockdown, and the lipid‐clearing effect of TRβ reactivation was nullified by PGC1α knockdown (Figure 4G,H; Figure S2A,B, Supporting Information). Triglyceride content analysis yielded similar results (Figure 4I), suggesting that TRβ may enhance lipid elimination through its interaction with coactivator PGC1α. In summary, TRβ suppressed ccRCC metastasis and malignant progression while promoting tumor cell “slimming” in a PGC1α‐dependent manner.

### 2.5 Deficiency of Mitochondrial Fusion and Downregulation of OPA1/MFN2 in ccRCC Tissues

**Figure 5:**
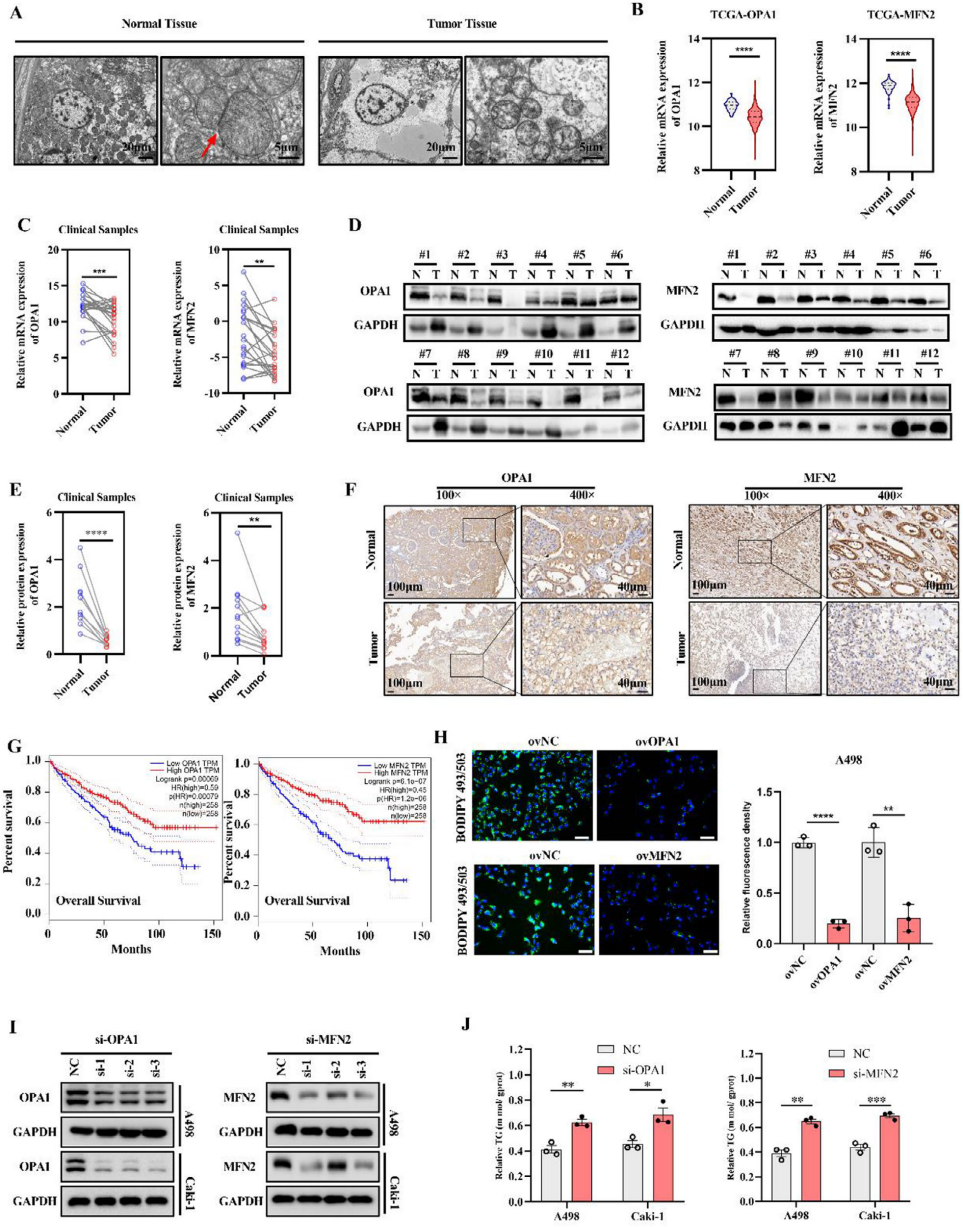
Mitochondrial fusion is impaired, and OPA1/MFN2 expression is reduced in ccRCC tissues. A) Mitochondrial fusion in ccRCC tissues visualized via electron microscopy. Red arrows indicate sites of mitochondrial fusion. B) OPA1 and MFN2 mRNA expression analyzed from the TCGA‐KIRC dataset. C) OPA1 and MFN2 mRNA levels in ccRCC tissues (*n* = 12). D,E) Protein levels of OPA1 and MFN2 in ccRCC tissues (*n* = 12). F) Immunohistochemical staining of OPA1 and MFN2 expression; dark brown staining indicates strong positivity. G) Kaplan‒Meier survival analysis assessed the correlation between OPA1 or MFN2 expression and overall survival in patients with ccRCC (log‐rank test). H) Bodipy staining was performed to assess lipid droplet accumulation in ccRCC cells overexpressing OPA1 or MFN2; statistical analysis was performed using a *t*‐test. I) Protein levels of OPA1 and MFN2 in ccRCC cell lines with negative control or knockdown. J) Triglyceride (TG) levels in the indicated cell lines (*n* = 3). Data are derived from at least three independent experiments (*p < 0.05, **p < 0.01, ***p < 0.001). OPA1 or MFN2‐specific overexpression lentivirus (ovOPA1 or ovMFN2); negative controls (ovNC); OPA1 and MFN2 siRNAs (si‐OPA1, si‐MFN2).

Mitochondria play a pivotal role in lipid droplet production and consumption through dynamic processes.^[^ ^33^, ^34^ ^]^ PGC‐1α is a key regulator of lipid metabolism and energy pathways, and is involved in maintaining mitochondrial fusion‐fission balance. Using electron microscopy (EM), we observed a mitochondrial dynamic change in mitochondrial morphology between ccRCC tissues and normal controls. A significant reduction in mitochondrial fusion was observed in ccRCC samples compared to normal tissues (**Figure** **5**A). Analysis of the TCGA‐KIRC database further revealed substantial downregulation of mitochondrial fusion markers, OPA1 and MFN2, in ccRCC (Figure 5B; Figure S3A, Supporting Information).

Analysis of clinical samples showed a significant reduction in both mRNA and protein levels of OPA1 and MFN2 (Figure 5C–E). This downregulation was corroborated by IHC analysis (Figure 5F; Figure S3B, Supporting Information). In addition, low levels of OPA1/MFN2 correlated with poor prognosis in ccRCC, whereas higher levels were associated with better outcomes (Figure 5G; Figure S3C, Supporting Information). We overexpressed OPA1 or MFN2 in RCC cell lines and observed a significant decrease in intracellular lipid droplets (Figure 5H; Figure S3D, Supporting Information). Conversely, inhibition of OPA1 or MFN2 led to increased lipid accumulation in A498 and Caki‐1 cells (Figure 5I,J). These results suggest that mitochondrial fusion deficiency, characterized by downregulation of MFN2/OPA1, contributes to lipid accumulation in ccRCC. Therefore, enhancing mitochondrial fusion may inhibit lipid buildup and improve ccRCC prognosis.

### 2.6 TRβ and PGC1α Coordinately Promote Mitochondrial Fusion

**Figure 6:**
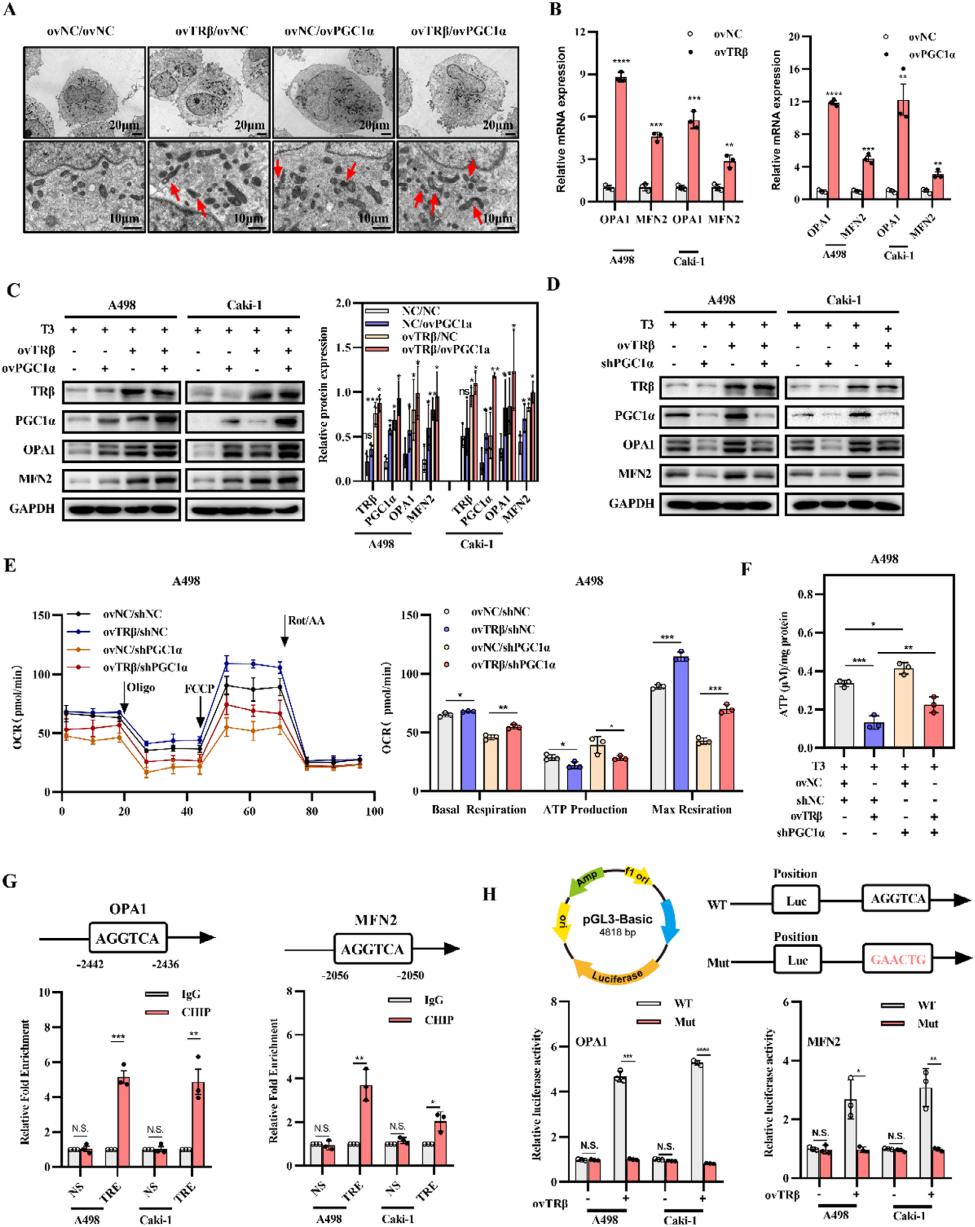
TRβ and PGC1α synergistically enhance mitochondrial fusion. A) Electron microscopy images showing mitochondrial fusion in ccRCC cells with TRβ and/or PGC1α overexpression. Red arrows indicate sites of mitochondrial fusion. B) Correlation analysis between TRβ expression and OPA1/MFN2 levels using TCGA‐KIRC data. B) OPA1 and MFN2 mRNA levels in ccRCC cells overexpressing TRβ and PGC1α. C) Western blot analysis of OPA1 and MFN2 protein levels in ccRCC cells overexpressing TRβ and/or PGC1α. D) OPA1 and MFN2 protein expression in ccRCC cells with TRβ overexpression and/or PGC1α knockdown. E) Real‐time oxygen consumption rate (OCR) in ccRCC cells with TRβ overexpression and/or PGC1α knockdown. Arrows indicate the time of inhibitor injections. Data are presented as mean ± SEM (*n* = 5 biological replicates). F) Intracellular ATP levels in ccRCC cells with TRβ overexpression and/or PGC1α knockdown. G) ChIP‐PCR analysis in ccRCC cell lines (*n* = 3). H) Dual‐luciferase reporter assay in ccRCC cells (*n* = 3, *t*‐test). Data represent at least three independent experiments (*p < 0.05, **p < 0.01, ***p < 0.001). TRβ overexpression lentivirus (ovTRβ); PGC1α knockout lentivirus (sh‐PGC1α); negative controls (ovNC).

EM was employed to observe morphological changes associated with TRβ/PGC1α‐mediated tumor cell “slimming” in A498 cells. Remarkably, restoration of either TRβ or PGC1α individually reduced mitochondrial fragmentation, whereas their combined reactivation significantly promoted mitochondrial fusion (**Figure** **6**A). Overexpression of TRβ or PGC1α increased OPA1 and MFN2 expression at the mRNA level (Figure 6B). Further investigation into the underlying biochemical mechanisms revealed a positive correlation between PGC1α and OPA1 (Pearson r = 0.3001, p < 0.0001), as well as MFN2 mRNA levels (Pearson r = 0.1728, p < 0.0001) based on TCGA data (Figure S4A, Supporting Information). Similarly, TRβ expression showed strong correlations with OPA1 (Pearson r = 0.3169, *p* < 0.0001) and MFN2 mRNA levels (Pearson r = 0.4306, *p* < 0.0001) (Figure S4B, Supporting Information), which were further validated in clinical samples (Figure S4C,D, Supporting Information). Combined reactivation of TRβ and PGC1α led to a synergistic enhancement of their expression (Figure 6C), whereas PGC1α knockdown reduced OPA1, MFN2, and UCP1 levels and partially reversed TRβ‐induced upregulation (Figure 6D; Figure S5A–C, Supporting Information). Mitochondrial respiratory function analysis using the Seahorse analyzer showed that reactivation of TRβ enhanced mitochondrial respiration but reduced ATP production, whereas inhibition of PGC1α partially reversed the TRβ‐induced reduction in ATP levels in renal cancer cells (Figure 6E). Consistently, direct ATP quantification showed that overexpression of TRβ reduced ATP production, and PGC1α inhibition partially reversed this effect (Figure 6F).

As TRβ functions as a transcription factor, a TRE, AGGTCA, was identified within the proximal promoter regions of OPA1 and MFN2 genes (Figure 6G). ChIP and luciferase experiments confirmed that TRβ transcriptionally activates OPA1 and MFN2 in ccRCC (Figure 6G,H). These results demonstrate that TRβ and PGC1α coordinately regulate OPA1/MFN2 transcription, thereby promoting mitochondrial fusion in ccRCC.

### 2.7 TRβ/PGC1α Suppressed ccRCC Progression via Tumor Cell “Slimming” and Mitochondrial Fusion In Vivo

**Figure 7:**
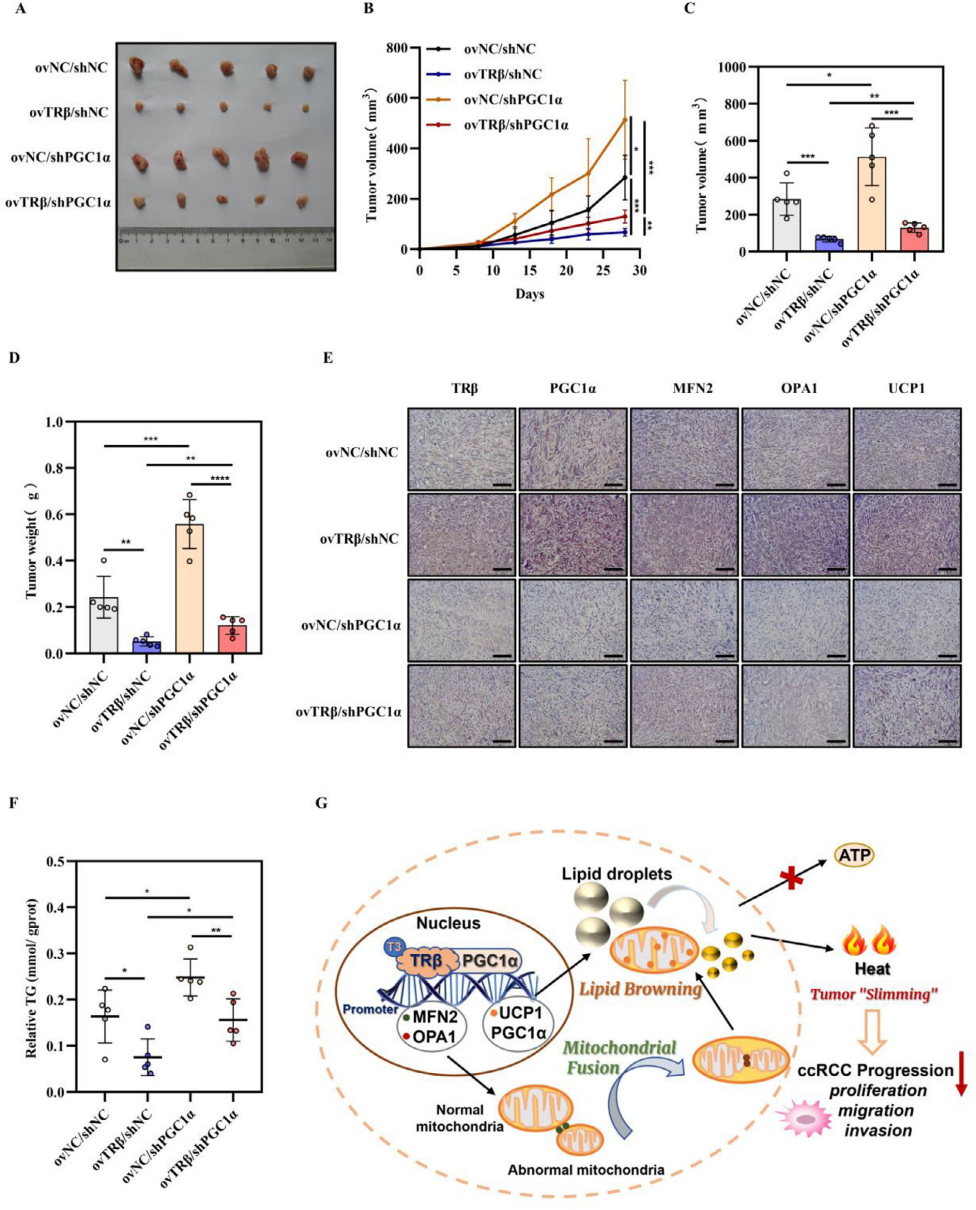
TRβ/PGC1α‐mediated tumor “slimming” and enhanced mitochondrial fusion reduce ccRCC progression in vivo. A) Representative images of xenografts formed from A498 cells with TRβ overexpression and/or PGC1α knockdown (*n* = 5). B) Tumor growth curves for xenografts across the four experimental groups (*n* = 5). C) Tumor volumes measured in xenografts from each group (*n* = 5). D) Tumor weights recorded in xenografts from the four groups (*n* = 5). E) Immunohistochemistry staining detecting TRβ, PGC1α, MFN2, OPA1, and UCP1 expression in tumor xenografts; dark brown staining indicates strong positivity. F) Relative triglyceride levels quantified in the four groups (*n* = 5). G) Schematic model illustrating the TRβ‐regulated tumor suppression mechanism affecting ccRCC growth and metastasis. Results are based on at least three independent experiments (*p < 0.05, **p < 0.01, ***p < 0.001). TRβ overexpression lentivirus (ovTRβ); PGC1α knockout lentivirus (sh‐PGC1α); negative controls (ovNC).

Building on the observation that TRβ/PGC1α promoted lipid browning and mitochondrial fusion in ccRCC cells, its functional role was further investigated in vivo. Xenograft tumor models were established in nude mice through subcutaneous injection of Caki‐1 cells with TRβ overexpression and/or PGC1α knockdown. Tumor size was measured every 3 days. The results indicated that PGC1α knockdown promoted tumor growth, as evidenced by increased volume and weight of the subcutaneous xenografts. Moreover, this tumor‐promoting effect counteracted the TRβ‐induced suppression of tumor growth (**Figure** **7**A–D), highlighting the collaborative regulation of tumor progression by TRβ and PGC1α. IHC analysis of xenograft tissues was subsequently performed to assess the protein expression of PGC1α, UCP1, OPA1, and MFN2. As anticipated, TRβ overexpression significantly elevated the protein levels of lipid browning marker UCP1 and mitochondrial fusion markers OPA1 and MFN2, whereas PGC1α knockdown resulted in the opposite effect and negated the TRβ‐induced enhancements in vivo (Figure 7E). In addition, Oil Red O staining revealed reduced lipid droplet accumulation in the TRβ overexpression group, while PGC1α knockdown reversed this inhibitory effect (Figure 7F). In summary, the TRβ/PGC1α complex significantly reduced tumor malignancy and enhanced lipid browning in vivo.

These results suggest that TRβ interacts with the coactivator PGC1α to transcriptionally activate lipid browning genes (PGC1α, UCP1) and mitochondrial fusion markers (OPA1, MFN2) by binding to the TREs in their proximal promoters. This coordinated regulation promotes lipid browning and mitochondrial fusion, facilitating tumor cell “slimming” and ultimately suppressing ccRCC progression (Figure 7G).

## 3 Discussion

This study provides compelling evidence that lipid accumulation and mitochondrial fusion defects contribute to the progression of ccRCC malignancies. Biochemical experiments identified the lipid browning genes PGC1α and UCP1, as well as mitochondrial fusion genes OPA1 and MFN2, as direct transcriptional targets of the TRβ‐PGC1α complex. Restoration of TRβ and PGC1α expression in ccRCC cell lines induced mitochondrial fusion, elevated UCP1 mRNA and protein levels, and inhibited lipid droplet formation. The significance of TRβ re‐expression was demonstrated by the reduction in lipid deposition, accompanied by a corresponding decrease in tumor growth. However, depletion of PGC1α in TRβ‐reconstituted cell lines reversed these effects, restoring the lipid deposition phenotype. Collectively, these findings suggest that addressing deficiencies in TRβ and PGC1α could offer a promising therapeutic strategy for RCC pathogenesis.

Energy metabolism plays a crucial role in tumor occurrence and progression.^[^ ^35^ ^]^ Glutamine metabolism, for example, contributes to the lung cancer immune microenvironment and influences responses to immunotherapy.^[^ ^36^ ^]^ Abnormal lipid metabolism is a hallmark of many tumors,^[^ ^37^, ^38^, ^39^, ^40^ ^]^ including phosphatidic acid metabolism^[^ ^41^ ^]^ and cholesterol metabolism.^[^ ^42^, ^43^ ^]^ Histologically, ccRCC is characterized by extensive lipid and glycogen deposits that fill the cytoplasm of tumor cells.^[^ ^44^, ^45^ ^]^ Recent studies suggest that this lipid accumulation results from impaired lipid breakdown, leading to a net increase in synthesis.^[^ ^46^, ^47^ ^]^ Therefore, strategies aimed at reversing this lipid deposition phenotype may offer effective interventions for renal cancer. Our previous studies demonstrated that PGC1α upregulates UCP1 and activates autophagy, promoting the breakdown of lipid droplets and enhancing lipid mobilization.^[^ ^10^ ^]^ UCP1‐mediated lipid browning consumed lipids to produce heat rather than ATP, representing a promising approach to counteract lipid accumulation disorders. In contrast, inhibition of PGC1α downregulated genes involved in mitochondrial biosynthesis, dynamics, and activity, and reduced mitochondrial membrane potential.^[^ ^48^ ^]^ PGC1α can also interact with cAMP response element binding protein and nuclear respiratory factors (NRFs), thereby regulating their transcriptional activities. Sirutin 1 functions upstream of PGC‐1α, which in turn recruits NRF2 to the promoter region of GPX4 and co‐activates GPX4 transcription as a coactivator rather than as a transcription factor.^[^ ^49^ ^]^ PGC1α acts as a transcriptional coactivator that increases the transcriptional activity of PPARG and TRs on the promoter of uncoupling protein genes. T3 induces adipose tissue browning in vitro,^[^ ^50^, ^51^ ^]^ with UCP1 expression in BAT requiring TRβ activation.^[^ ^52^ ^]^ One of the most striking findings of this study is that PGC1α functions as a TRβ coactivator, directly activating the transcription and expression of UCP1 and PGC1α, thus driving lipid browning and metabolic alterations in ccRCC. Nevertheless, this study has certain limitations: whether PGC1α may activate other lipid metabolic pathways beyond UCP1‐mediated thermogenesis in ccRCC requires further investigation.

Hormones and their receptors play critical roles in tumor progression.^[^ ^53^, ^54^, ^55^ ^]^ In certain cancers, including breast cancer, TR activity can promote cell proliferation, whereas in others, such as liver cancer, it may function as a tumor suppressor.^[^ ^56^ ^]^ This study confirms the tumor‐suppressive role of TRβ in ccRCC, establishing its significance as a key prognostic marker through ROC analysis and other assessments. Moreover, the findings underscore the distinct function of hormone‐related receptor signaling pathways in tumor development.

T3 has recently emerged as a potent antitumor agent, and the T3/TRβ axis exerts a unique inhibitory effect on tumor growth in ccRCC. Our results demonstrate that ccRCC remains responsive to T3 treatment, aligning with previous reports showing that T3 can interfere with tumor development. Moreover, impaired thyroid hormone function, particularly reduced fT3/fT4 ratio, is a strong prognostic factor for worse prognosis of metastatic RCC.^[^ ^57^ ^]^ Recently, new thyroid hormone analogs, particularly selective TRβ agonists, have gained attention for treating nonalcoholic fatty liver disease, with initial clinical trial results showing promise.^[^ ^58^ ^]^ These TRβ agonists leverage the effects of thyroid hormone on lipid metabolism, offering potential therapeutic applications.^[^ ^59^ ^]^ This study proposes that treating ccRCC with TRβ agonists could be beneficial by targeting lipid metabolism through TRβ‐mediated lipid browning and mitochondrial fusion pathways. Nevertheless, their impact on tumor lipid metabolism requires thorough investigation, and the potential effects of systemic TRβ agonist administration on other organs need further clarification. Future studies should validate this mechanism in more complex orthotopic or PDX models and delve deeper into this combination therapy strategy.

The role of mitochondrial dynamics in cellular metabolism, particularly lipid metabolism, remains incompletely understood. For instance, BAT involution is characterized by reduced mitochondrial mass and increased lipid deposition.^[^ ^60^ ^]^ Studies have shown that dysfunction in mitochondrial fusion impairs fatty acid oxidation, leading to heightened fat accumulation. This study identifies impaired mitochondrial fusion in RCC, characterized by significant downregulation of the fusion markers OPA1 and MFN2, which correlates with poor prognosis. Depletion of OPA1 or MFN2 induced BAT whitening.^[^ ^61^ ^]^ Restoration of OPA1/MFN2‐mediated mitochondrial fusion appears to reestablish mitochondrial homeostasis, augment functional capacity, and improve energy metabolism, thereby facilitating UCP1‐dependent lipid clearance in RCC cells. Enhanced fusion may further promote fatty acid trafficking toward mitochondria, potentially via regulated mitochondrial‐lipid droplet interactions, culminating in UCP1‐mediated energy dissipation. However, the precise mechanisms underlying these processes require further elucidation. In summary, this study uncovered a link between mitochondrial fusion and lipid metabolism in renal cancer. In addition, this study demonstrated that the T3/TRβ axis promotes “tumor slimming” and suppresses ccRCC progression through PGC1α/UCP1‐mediated lipid browning and OPA1/MFN2‐mediated mitochondrial fusion. During this process, lipid browning reduces lipid accumulation in ccRCC cells without generating ATP as an energy source. These findings highlight that PGC‐1α can recruit TRβ to the promoter region of UCP1, PGC1α, OPA1, and MFN2, and co‐activate their transcription. Reactivation of the T3/TRβ/PGC1α axis thus holds therapeutic potential for inducing lipid elimination and promoting tumor “slimming.” Thus, combining TRβ and PGC1α analogs could serve as a valuable therapeutic strategy for ccRCC. Although TRβ downregulation in certain tumors may limit antitumor efficacy, a subset of cancer cells may retain sufficient TRβ expression to respond to agonist therapy. TRβ agonists can act through multiple mechanisms: enhancing residual TR signaling to promote mitochondrial fusion or UCP1‐mediated lipid browning; targeting TRβ‐expressing stromal or immune cells within the tumor microenvironment to exert indirect effects; or potentially upregulating TRβ through unidentified feedback pathways. Furthermore, gene editing targeting TRβ/PGC1α activation or expression represents a promising therapeutic strategy for ccRCC.

In conclusion, this study uncovered a previously unrecognized mechanism involving TRβ and PGC1α in renal cancer, linking hormone receptor proteins to lipid browning during cancer progression. Although the broader implications of this molecular mechanism for other tumor types remain unclear, these findings may provide insight into similar metabolic pathways in other cancers, potentially extending to further cancer research and treatment strategies.

## 4 Experimental Section

### Human ccRCC and Paired Adjacent Specimens

Human ccRCC tumor samples were obtained from patients undergoing surgical resection at Wuhan Union Hospital, Tongji Medical College, Wuhan, China. The study was approved by the Ethics Committee of Wuhan Union Hospital (Approval No. IEC‐072). All procedures were conducted in accordance with ethical guidelines for research involving human subjects. Informed consent was obtained from all participants. Immediately after resection, fresh tumor and adjacent tissues were snap‐frozen in liquid nitrogen.

### Animals and Xenograft Studies

All animal experiments were conducted with approval from the Animal Care and Use Committee of Tongji Medical College, Huazhong University of Science and Technology (S175). Male mice, aged 6–8 weeks, were purchased from Beijing HFK Bioscience (Beijing, China) and housed five per cage. For experimental purposes, mice were randomly assigned to different groups. To establish an in vivo xenograft model, 1 × 10^6^ Caki‐1 cells were injected subcutaneously into the flanks of the mice. Once tumors reached a palpable size (≈100 mm^3^), the mice were administered either a T3‐supplemented diet (2.5 µg d^−1^) or a vehicle (phosphate‐buffered saline [PBS]) via oral gavage for 30 days. The doses of T3 administered in this study were selected based on previous findings, which demonstrated that these levels do not adversely affect animal health.^[^ ^62^ ^]^ Tumor size was monitored every 5‒7 days with calipers, and tumor weight was recorded upon sacrifice, 4‒6 weeks post‐implantation. Tissue samples were collected for IHC and protein analysis. All animal procedures complied with all relevant ethical regulations.

### Cell Culture and Reagents

The 786‐0, A498, Caki‐1, and HK2 cell lines were obtained from the American Type Culture Collection (Manassas, VA), and the cell lines were contamination‐free. HK2, a renal proximal tubular epithelial cell, was derived from normal human kidney tissue. 786‐O was derived from primary ccRCC, whereas A498 was isolated from the kidney cancer tissue. Caki‐1 was derived from skin metastatic cells of human ccRCC. HK2 cells were used as a control for comparison with primary and metastatic ccRCC cells. Cells were cultured in DMEM supplemented with 10% fetal bovine serum and 1% penicillin‐streptomycin, and maintained at 37 °C in a 5% CO_2_ incubator.

Oil Red O dye was sourced from Wuhan Servicebio Technology, and the triglyceride assay kit (A110‐1‐1) was provided by Nanjing Jiancheng Bioengineering Institute. Liothyronine (T3) was purchased from MedChemExpress (MCE). Cells were treated with or without 100 nm T3 for 48 h, with doses and timeframes based on previous studies.^[^ ^63^, ^64^ ^]^

### Gene Expression and Silencing

TRβ, PGC1α, OPA1, and MFN2 overexpression lentivirus (ovTRβ, ovPGC1α, ovOPA1, and ovMFN2), PGC1α knockout lentivirus (sh‐PGC1α), and respective negative controls (ovNC) were obtained from GeneChem (Shanghai, China). To generate stable cell lines, cells were transduced with these lentiviruses using HitransG P (20 µL mL^−1^, GeneChem) and incubated for 72 h, followed by selection with 2 mg mL^−1^ puromycin for 3 days. OPA1 and MFN2 siRNAs (si‐OPA1, si‐MFN2) were purchased from GenePharma (Suzhou, China) (Table S1, Supporting Information). Cells were transduced with siRNAs using Lipofectamine RNAiMAX (Thermo, USA) based on the protocols of the manufacturer. Knockdown, overexpression, and gene silencing efficiencies were confirmed by qPCR and Western blotting.

### Cell Proliferation and Colony Formation Assays

Cell proliferation assay was conducted following a previously established protocol.^[^ ^11^ ^]^ Briefly, 2000 cells were seeded into 96‐well plates, and proliferation rates were assessed using the MTS assay, adhering to the instructions of the manufacturer. For colony formation assays, 1000 cells were plated in six‐well plates and incubated for 2 weeks. Surviving colonies were then stained with 0.05% crystal violet. To assess the effect of T3, a similar protocol was used, except that ccRCC cells were additionally cultured in media containing T3 (100 nm) or without T3, with experiments conducted as previously described.

### Transwell Assays

Cell migration assays were performed with slight modifications. A498 and Caki‐1 cells were pretreated in a serum‐free medium for 24 h before seeding into the upper chamber of a Corning insert. After 24 h, cells on the lower membrane surface were fixed with 100% methanol, stained with 0.05% crystal violet, photographed, and counted using phase‐contrast microscopy. For invasion assays, the same protocol was applied; however, cells were allowed to invade through Matrigel (Thermo Fisher Scientific), and Transwell assays were completed as described.

### Oil Red O Staining and Triglyceride Content

Oil Red O staining and triglyceride content assays were performed as previously described.^[^ ^65^ ^]^ When cells reached ≈40% confluency, they were rinsed with PBS and fixed in 4% paraformaldehyde for 30 min. Subsequently, cells were stained with 1 mL of Oil Red O solution (Wuhan Servicebio Technology) at room temperature for 30 min. Triglyceride content was measured using the Triglyceride Assay Kit (Nanjing Jiancheng Bioengineering Institute) based on the protocol of the manufacturer.

### Transmission Electron Microscopy

Fresh tissue samples were fixed in 2.5% phosphate‐buffered glutaraldehyde, followed by post‐fixation in 1% phosphate‐buffered osmium tetroxide. Samples were embedded, sectioned, and stained with uranyl acetate and lead citrate before visualization using an H‐7650 transmission electron microscope (Hitachi, Japan).

### RNA Isolation and qRT‐PCR

Total RNA was extracted from tissues and cells using TRIzol reagent (Thermo, USA), followed by reverse transcription. Real‐time PCR was performed with SYBR Green mix (Thermo, USA), with GAPDH serving as the normalization control. Primers for qRT‐PCR were obtained from Sangon Biotech (Shanghai) (Table S2, Supporting Information).

### ChIP‐qPCR

ChIP‐qPCR was performed using the SimpleChIP Enzymatic Chromatin IP Kit (Agarose Beads, #9002, CST) with TRβ antibodies and native IgG as a control, following the protocol of the manufacturer. DNA was analyzed by qPCR, with primers for ChIP analysis obtained from Sangon Biotech (Shanghai) (Table S2, Supporting Information).

### Western Blotting and IHC

Western blotting assays were conducted as previously described.^[^ ^66^ ^]^ Primary antibodies used included PGC1α (1:1000, Abcam), UCP1 (1:1000, Abcam), OPA1 (1:1000, Abclonal), and MFN2 (1:1000, Abclonal). GAPDH (1:1000, Abclonal) served as a loading control. Secondary antibodies were purchased from Proteintech. TRβ antibody (1:1000, sc‐398007) was obtained from Santa Cruz Biotechnology, Inc. IHC was performed as previously described.^[^ ^67^ ^]^ Protein levels were assessed based on staining intensity, with dark brown indicating strong positivity.

### Co‐Immunoprecipitation

A498 cells were transfected with various plasmids for 72 h and cultured in media containing T3 (100 nm). Subsequently, cells were lysed using an ice‐cold Triton‐lysis buffer. The supernatants from these lysates were subjected to immunoprecipitation with the appropriate primary antibodies, followed by overnight rotation at 4 °C. Protein A/G PLUS‐Agarose (Santa Cruz, CA, USA) was then added and incubated for 2 h at room temperature. The immunoprecipitates were washed three times with PBST and subsequently analyzed via Western blotting.

### IHC Staining Assays

Paraffin‐embedded ccRCC tumor sections (4 µm thick) were deparaffinized, blocked, and incubated with primary antibodies overnight at 4 °C. After incubation with secondary antibodies for 2 h at room temperature, 3,3′‐Diaminobenzidine Tetrahydrochloride was added, and the sections were counterstained with hematoxylin to visualize the staining.

### Luciferase Assays

For luciferase reporter assays, cells were seeded in 48‐well plates, and 500 ng of complementary DNA was co‐transfected with Lipofectamine 2000 (Invitrogen). At 72 h post‐transfection (when cell fusion reached 90%), cells were collected, and luciferase activity was measured using the Dual‐Luciferase Assay reagent (Promega, E1910) based on the instructions of the manufacturer.

### Bioinformatics Analysis

Lipid metabolism‐related gene datasets were retrieved from the GENECARDS database, whereas hormone‐ and transcription factor‐related gene datasets were acquired from the MSigDB database. Clinical cohort data and gene expression profiles were sourced from cBioPortal for the TCGA‐KIRC dataset. Gene expression profiles in normal tissues were obtained from the Human Protein Atlas database. Pathway analyses were performed using gene set enrichment analysis.

### Oxygen Consumption Rate (OCR) Assay

Mitochondrial respiratory parameters were quantified through OCR profiling using an XFe24 analyzer (Agilent Technologies). Prior to measurements, XF96 microplates (103793‐100) were seeded with 5 × 10⁴ cells per well and maintained until adherence. Sensor cartridges were hydrated with Seahorse XF96 calibrant (100840‐100) and subjected to overnight incubation at 37 °C in a non‐CO_2_ environment. Before assay initiation, the medium was replaced with pre‐warmed Seahorse XF DMEM basal medium (103575‐100) enriched with energy substrates (glucose 103577‐100, sodium pyruvate 103578‐100, and glutamine 103579‐100), followed by 60 min equilibration under non‐CO_2_ conditions. Following instrument calibration, real‐time OCR measurements were recorded starting with a baseline, followed by automated delivery of mitochondrial perturbagens (oligomycin A, FCCP, rotenone/antimycin A, each 1 µm) from the Cell Mito Stress Test Kit (103015‐100). The experimental design incorporated three‒five replicate measurements for each biological condition.

### ATP Assay

ATP levels in tumor cells were quantified using a firefly luciferase‐based ATP assay kit (Beyotime, China) based on the instructions of the manufacturer. Briefly, harvested cell pellets were lysed and centrifuged to obtain soluble fractions. The resulting supernatants were transferred to a 96‐well plate and incubated with the ATP detection working solution. Standard curves were constructed in parallel for absolute quantification. All ATP measurements were normalized to total protein concentrations, determined by the bicinchoninic acid method.

### Bodipy

Cells were seeded in 96‐well plates and allowed to adhere. At ≈50% confluence, the cultures were incubated with a serum‐free DMEM solution containing 2 µm BODIPY 493/503 (HY‐W090090, MCE, USA) for 30 min at 37 °C. Following staining, nuclei were counterstained with DAPI (C0060, Solarbio, China). Fluorescence images were acquired at 200× magnification using a fluorescence microscope.

### Statistical Analysis

Statistical analyses were conducted using Microsoft Excel 2013, GraphPad Prism v.5.00, and SPSS Statistics 22.0 (IBM SPSS). All experiments were conducted at least three times, and results were presented as mean ± S.E. Comparisons between groups were performed using unpaired two‐sided Student's *t*‐tests or ANOVA. Overall and disease‐free survival times were calculated from the time of diagnosis, and Cox proportional hazards regression was used to adjust for survival‐related risk factors. ROC curves were used to assess clinical diagnostic value. Correlation analyses were conducted using the Pearson correlation coefficient. A *p*‐value <0.05 was considered statistically significant, with significance levels indicated as follows: *, *p* < 0.05; **, *p* < 0.01; ***, *p* < 0.001; ****, *p* < 0.0001.

## Conflict of Interest

The authors declare no conflict of interest.

## Author Contributions

X.G.M., T.X.Y., F.L., and W.Q.L. contributed equally to this work. W.X. and X.P.Z. designed the study. X.G.M., T.X.Y., F.L., and W.Q.L. performed the experiments. H.M.Y. and W.X. analyzed the data. W.X. and X.P.Z. wrote the paper.

## Ethics Approval and Consent to Participate

The experimental protocols and study procedures were approved by the Institutional Review Board of Union Hospital, affiliated with Tongji Medical College, Huazhong University of Science and Technology. All animal experiments were conducted in accordance with the guidelines of the Institutional Animal Use and Care Committee of Tongji Medical College, Huazhong University of Science and Technology.

## Consent for Publication

All authors contributed to the study and have reviewed and approved the final version of the submitted manuscript.

## Supporting information



Supporting Information

## Data Availability

The data that support the findings of this study are available from the corresponding author upon reasonable request.
