# Supplementary material for: Thyroid Hormone Receptor β1 and PGC1α Coordinately Regulate OPA1/MFN2‐Mediated Mitochondrial Fusion and UCP1‐Mediated Lipid Browning in ccRCC
Source: Adv Sci (Weinh). 2025 Nov 9;13(5):e08571. doi: 10.1002/advs.202508571 (PMC12849942; doi:10.1002/advs.202508571)
Supplement: Supplementary file 1 — Supporting Information [file ADVS-13-e08571-s001.docx]

**Thyroid hormone receptor β1 (TRβ) and PGC1α coordinately regulate OPA1/MFN2-mediated mitochondrial fusion and UCP1-mediated lipid browning in ccRCC**

**Xiangui Meng****^1,2,3#^, Tiexi Yu^1,2,3#^, Fang Lv^1,2,3#^, Weiquan Li^1,2,3#^, Hongmei Yang^4*^, Xiaoping Zhang^1,2,3*^,** **Wen Xiao****^1,2,3*^**

1 Department of Urology, Union Hospital, Tongji Medical College, Huazhong University of Science and Technology, Wuhan 430022, China

2 Shenzhen Huazhong University of Science and Technology Research Institute, Shenzhen 518000, China

3 Institute of Urology, Tongji Medical College, Huazhong University of Science and Technology, Wuhan 430022, China

4 Department of Pathogenic Biology, School of Basic Medicine, Tongji Medical College, Huazhong University of Science and Technology, Wuhan, 430030, China.

#XGM, TXY, FL, WQL contributed equally to this work

Corresponding to:

Xiaoping Zhang, Department of Urology, Union Hospital, Tongji Medical College, Huazhong University of Science and Technology, 1277 Jiefang Avenue, Wuhan, Hubei Province, 430022(China); FAX number: 85776343; Email: [xzhang@hust.edu.cn](mailto:xzhang@hust.edu.cn)

Hongmei Yang, Department of Pathogenic Biology, School of Basic Medicine, Tongji Medical College, Huazhong University of Science and Technology, Wuhan, 430030, China. Email: hyang@hust.edu.cn.

Wen Xiao, Department of Urology, Union Hospital, Tongji Medical College, Huazhong University of Science and Technology,1277 Jiefang Avenue, Wuhan, Hubei Province, 430022(China); E-mail: wxuro20@hust.edu.cn

**Supplementary**

**
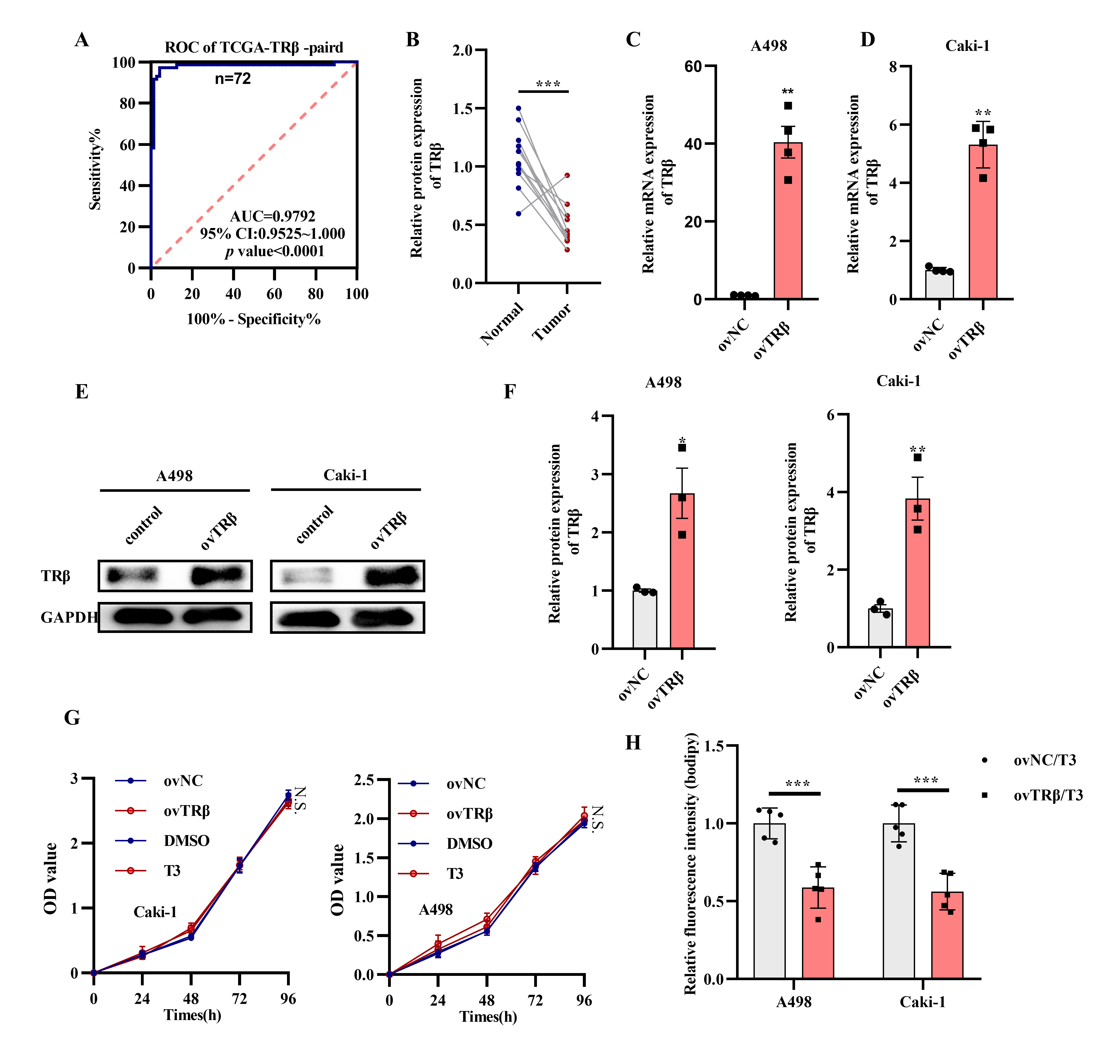
**

**sFigure 1. TRβ downregulation in ccRCC correlates with poor prognosis.** (A) ROC analysis of TRβ expression in ccRCC versus adjacent normal tissues using TCGA-KIRC data (t-test). (B) Protein expression of TRβ in ccRCC tissues and adjacent normal tissues (n = 12, t-test). (C-D) TRβ mRNA expression in ccRCC cell lines overexpressing TRβ (n = 3). (E-F) Protein levels of TRβ in ccRCC cell lines with TRβ overexpression (n = 3). (G) CCK8 proliferation assays of TRβ-overexpressing cell lines without T3 treatment (n = 4). (H) TRβ-restore revealed a decreased lipid accumulation in Caki-1 and A498 cell lines with bodipy staining(n = 5).TRβ overexpression lentivirus (ovTRβ), negative controls (ovNC).


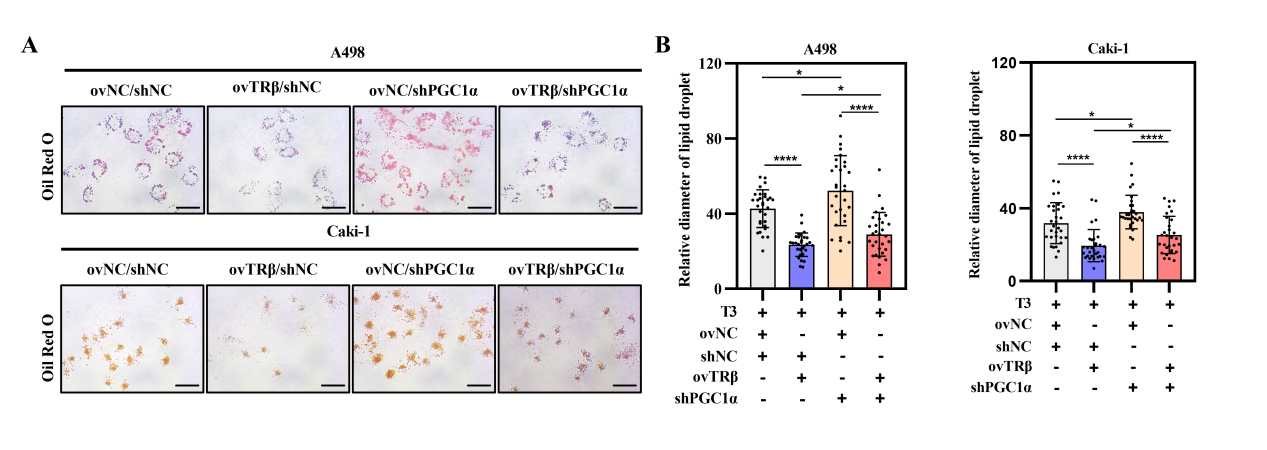
**sFigure 2.** The lipid-clearing effect of TRβ reactivation was nullified by PGC1α knockdown. (A) and (B) TRβ-restore revealed a marked decrease in lipid accumulation, and the lipid-clearing effect of TRβ reactivation was nullified by PGC1α knockdown in renal cancer cells with ORO staining.TRβ overexpression lentivirus (ovTRβ), PGC1α knockout lentivirus (sh-PGC1α), negative controls (ovNC)


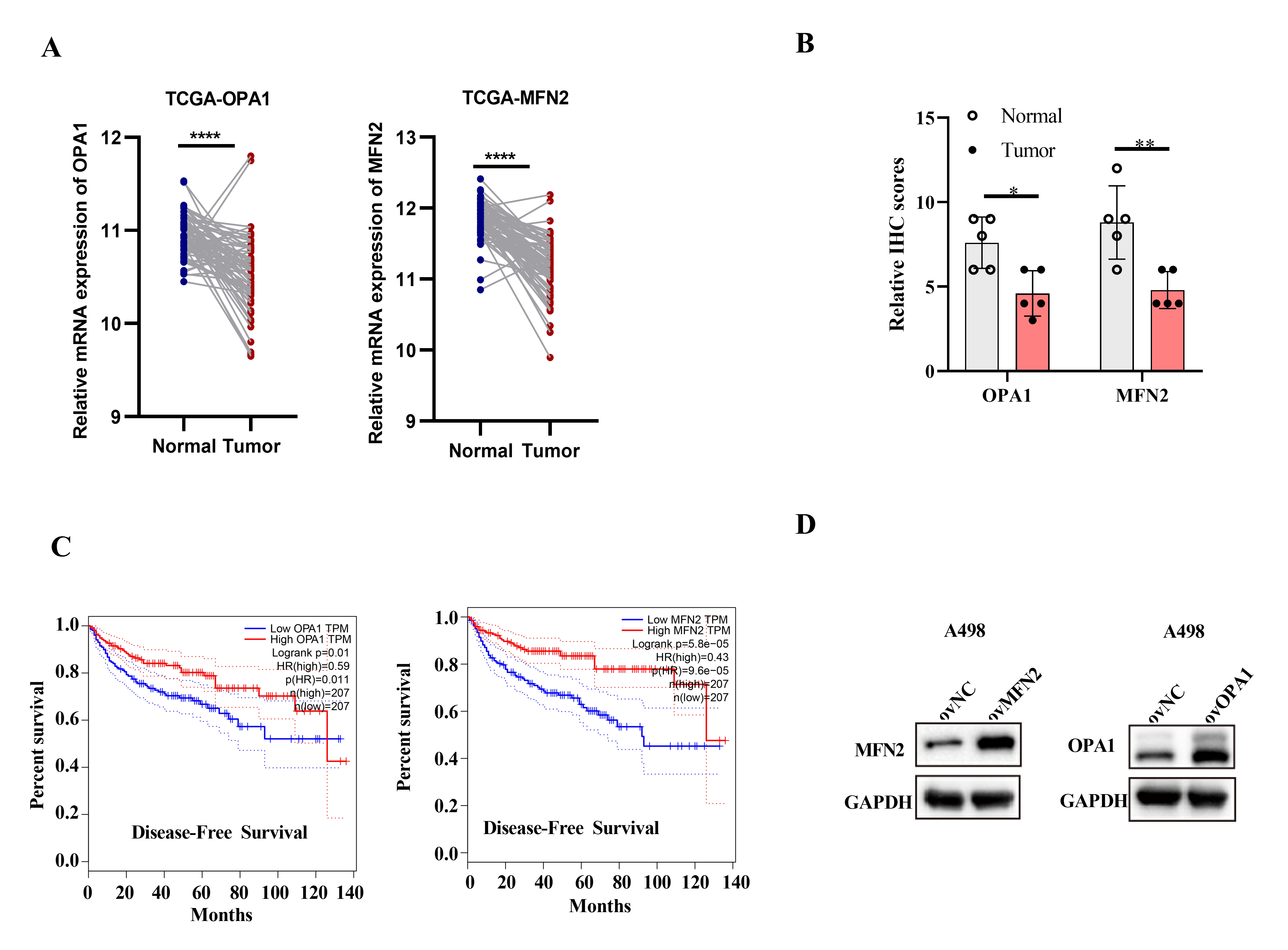


**sFigure 3. The role of OPA1/MFN2 in ccRCC.** (A) OPA1 and MFN2 mRNA expression levels in the TCGA-KIRC dataset. (B) OPA1 and MFN2 protein expression levels in clinical samples. (C) Low levels of OPA1/MFN2 correlated with poor DFS prognosis in ccRCC. (D) Overexpression of OPA1 or MFN2 in renal cancer cells.OPA1 or MFN2-specific overexpression lentivirus (ovOPA1 or ovMFN2), negative controls (ovNC)


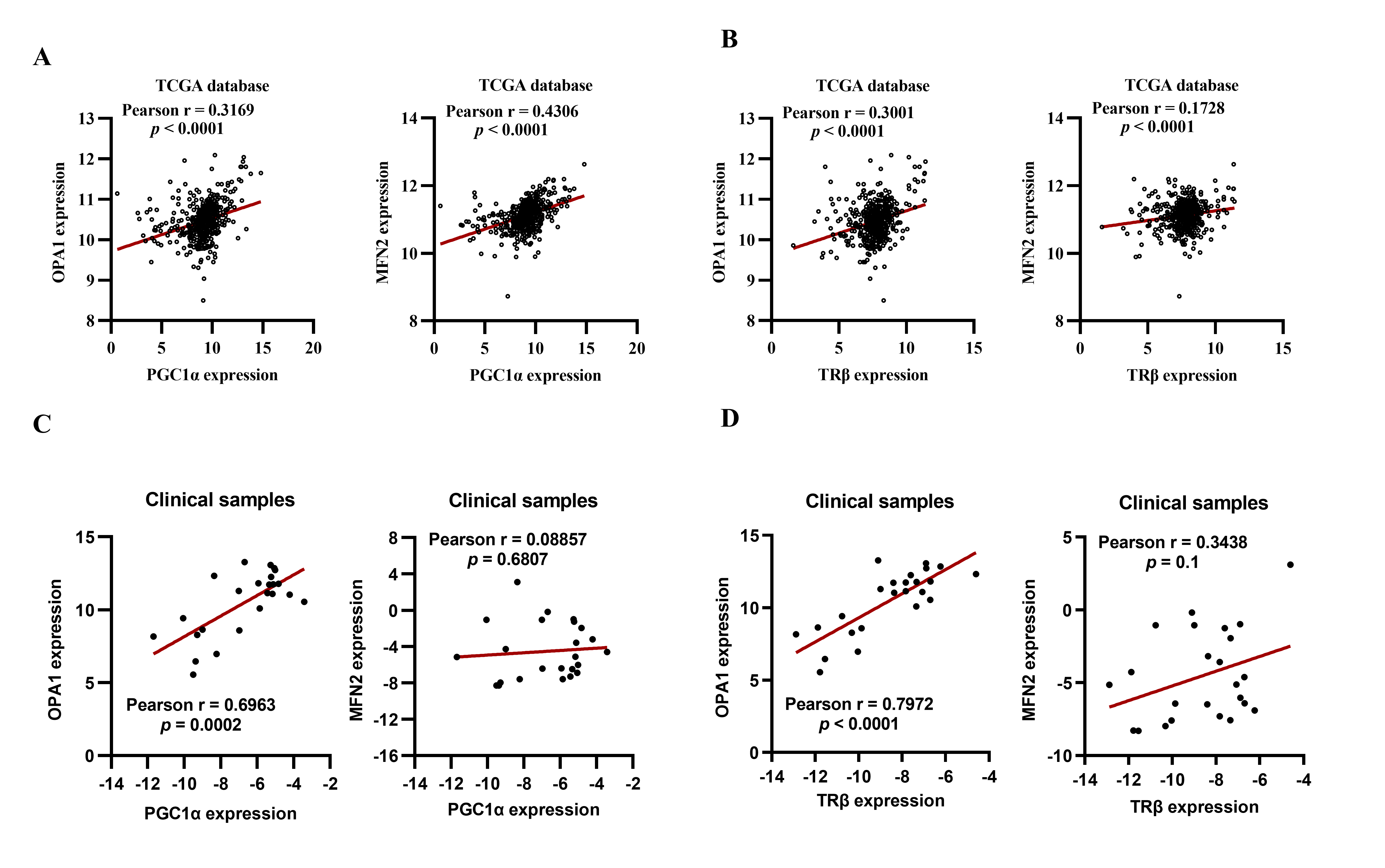


**sFigure 4. The** correlation **of TRβ/PGC1α and OPA1/MFN2 in ccRCC .** (A-B) Correlation analysis between TRβ/PGC1α and mitochondrial fusion markers in the TCGA-KIRC dataset. (C-D) Correlation analysis between TRβ/PGC1α and mitochondrial fusion markers in clinical samples.

**
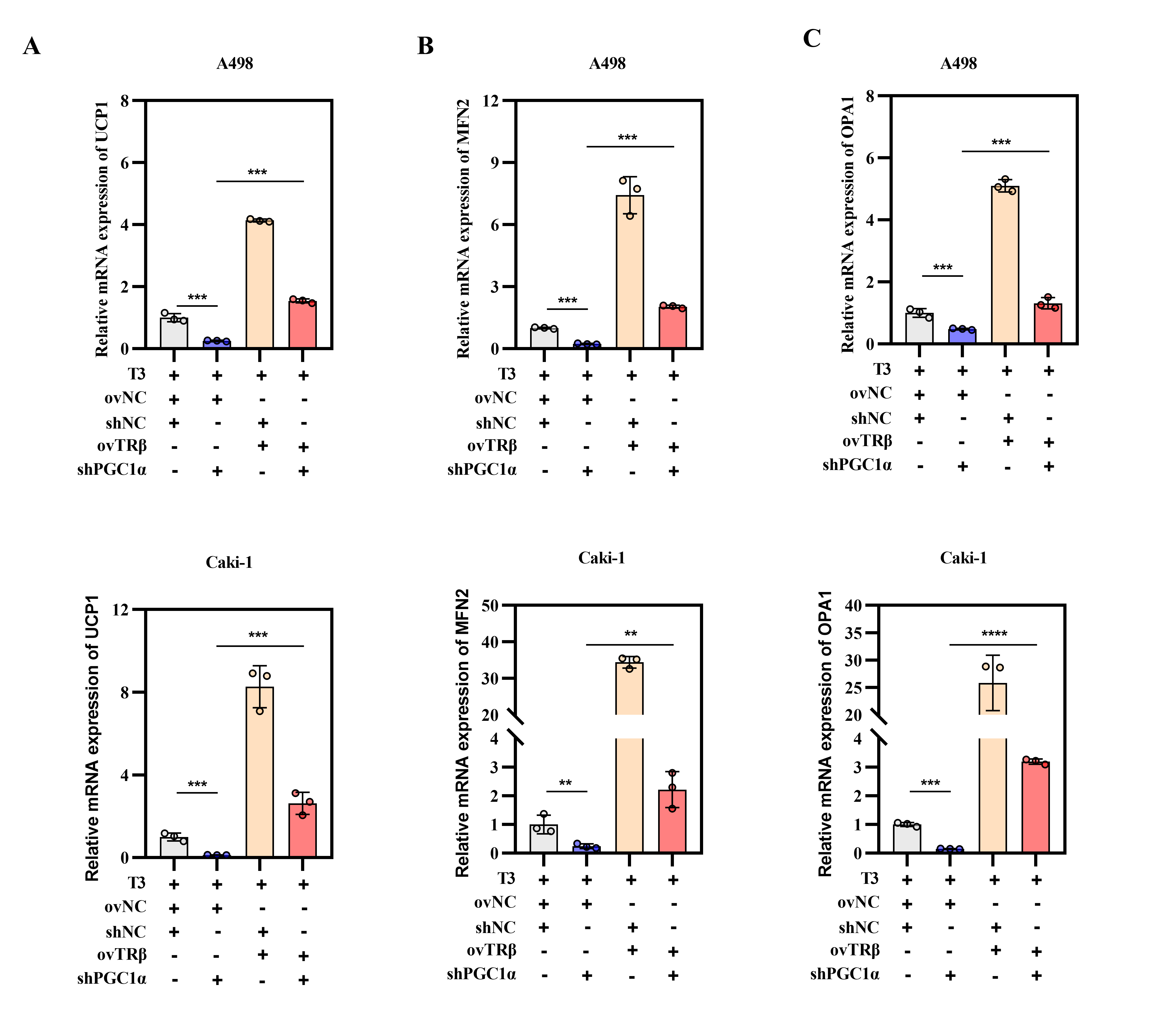
**

**sFigure 5.** Role of TRβ/PGC1α in UCP1 and mitochondrial fusion protein. (A)-(C)TRβ reactivation led to a synergistic enhancement in UCP1 and mitochondrial fusion protein, while PGC1α knockdown reduced their levels and partially reversed the TRβ-induced upregulation (n=3). TRβ overexpression lentivirus (ovTRβ), PGC1α knockout lentivirus (sh-PGC1α), negative controls (ovNC)

# Supplementary Table 1

**The sequences of siRNA fragments**

| **Primer names** | **Sequences (5′- 3′)** | |
| --- | --- | --- |
| si-TRβ-1 (si-1) | CCUUACAGCCUGGGACAAATT | UUUGUCCCAGGCUGUAAGGTT |
| si-TRβ-2 (si-2) | CCACUUGGACUAGCUCAAUTT | AUUGAGCUAGUCCAAGUGGTT |
| si-TRβ-1(si-1) | GGUAUCACUACCGCUGUAUTT | AUACAGCGGUAGUGAUACCTT |
| si-OPA1-2 (si-1) | GCCUGACAUUGUGUGGGAATT | UUCCCACACAAUGUCAGGCTT |
| si-OPA1-2 (si-2) | GCAUGGCUCCUGACACAAATT | UUUGUGUCAGGAGCCAUGCTT |
| si-OPA1-3(si-3) | GCUAUCACCGCAAAUACUUTT | AAGUAUUUGCGGUGAUAGCTT |
| si-MFN2-1 (si-1) | CCAUGAGGCCUUUCUCCUUTT | AAGGAGAAAGGCCUCAUGGTT |
| si-MFN2-2 (si-2) | GCUCUUGGCUCAAGACUAUTT | AUAGUCUUGAGCCAAGAGCTT |
| si-MFN2-3 (si-3) | CCAGUAGUCCUCAAGGUUUTT | AAACCUUGAGGACUACUGGTT |

# Supplementary Table 2

**Gene primers of qRT-PCR**

| **Gene** | **Forward primer (5′‐3′)** | **Reverse primer (5′‐3′)** |
| --- | --- | --- |
| GAPDH | GAGTCAACGGATTTGGTCGT | GACAAGCTTCCCGTTCTCAG |
| PGC1α | AGCCTCTTTGCCCAGATCTT | GGCAATCCGTCTTCATCCAC |
| TRβ | AGACGCCATCTTTGACCTGG | GTGTCACGTGGTGTTTTCGG |
| UCP1 | GCGGATGAAACTCTACAGCG | TTGATTCCGTGGAGATGGCT |
| OPA1 | TGTGATTGAAAACATCTACCTTCCA | TTTAAGCTTGATATCCACTGTGGTGT |
| MFN2 | CACAAGGTGAGTGAGCGTCT | AGGAAGCTGGTACAACGCTC |
